# Reflectance Confocal Microscopy Integrated With Dermoscopy in a Real‐World Tertiary Referral Cohort: Diagnostic Agreement, Management Outcomes, and Number Needed to Excise in 1057 Lesions

**DOI:** 10.1111/ijd.70601

**Published:** 2026-07-28

**Authors:** Luca Ambrosio, Claudio Conforti, Giovanni Di Lella, Carmen Cantisani, Francesca Svara, Federico Garbarino, Alessia Paganelli, Camilla Chello, Caterina Longo, Steven Paul Nisticò, Giovanni Pellacani

**Affiliations:** ^1^ IDI‐IRCCS, Dermatological Research Hospital Rome Italy; ^2^ Dermatology Unit, Department of Medical and Cardiovascular Sciences Sapienza University of Rome Rome Italy; ^3^ Department of Life Science, Health and Health Professions Link University of Rome Rome Italy; ^4^ Department of Dermatology University of Modena and Reggio Emilia Modena Italy

**Keywords:** clinical management, cutaneous neoplasms, dermoscopy, melanoma, number needed to excise, reflectance confocal microscopy

## Abstract

**Background:**

Accurate non‐invasive assessment of cutaneous lesions requires integration of diagnostic information with real‐world management outcomes. Reflectance confocal microscopy (RCM) is increasingly used as a second‐level tool after dermoscopy, but evidence linking diagnostic agreement, histological verification, and management across melanocytic and non‐melanocytic lesions remains limited.

**Methods:**

This retrospective observational study included 886 patients with 1057 cutaneous lesions evaluated by combined dermoscopy and RCM at a tertiary referral center between October 2023 and September 2024. Lesions were classified as benign melanocytic, malignant melanocytic, benign non‐melanocytic, or malignant non‐melanocytic. RCM–dermoscopy agreement was assessed using observed agreement and Cohen's kappa; chi‐square testing evaluated diagnostic association. Histology‐based performance analyses were restricted to histologically confirmed lesions.

**Results:**

Among 931 lesions with both classifications available, observed agreement was 37.8%, with fair agreement by Cohen's kappa (*κ* = 0.211; 95% confidence interval [CI], 0.172–0.251). Diagnostic distributions were significantly associated (*χ* ^2^ = 571.3, df = 9, *p* < 0.001), with substantial discordance, particularly within melanocytic categories. Histopathological confirmation was available for 225 lesions (21.3%), indicating verification bias. In the dedicated melanocytic excision subset, 162 excised lesions yielded 41 melanomas, resulting in a numbers needed to excise (NNE) value of 3.95. Malignant non‐melanocytic detection yield was 39.0% among histologically confirmed excised non‐melanocytic lesions.

**Conclusions:**

In this real‐world tertiary referral cohort, RCM integrated with dermoscopy supported lesion stratification and management across diagnostic categories. The significant association but fair agreement between modalities highlights their complementary role. Diagnostic performance estimates should be interpreted within the histology‐confirmed subset and in light of verification bias.

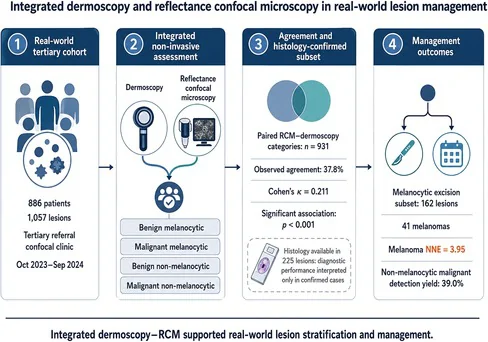

## Summary

Reflectance confocal microscopy (RCM) is a non‐invasive imaging technique that enables in vivo visualization of skin lesions at near‐histological resolution. When used together with dermoscopy, it may provide additional information to assess lesions that remain clinically or dermoscopically equivocal. In this retrospective real‐world study, we analyzed 1058 cutaneous lesions from 886 patients evaluated at a tertiary referral center using combined dermoscopy and RCM. We investigated the relationship between dermoscopic and confocal diagnoses, clinical management patterns, the number of excisions required to identify one melanoma, and the influence of anatomical site on diagnosis and device utilization. Among 162 excised melanocytic lesions, 41 were confirmed as melanomas, corresponding to a number needed to excise of 3.95. Surgical excision rates decreased over time, whereas clinical monitoring without advanced imaging increased. The anatomical site significantly influenced both the diagnostic distribution and the selection of the confocal imaging device. These findings provide real‐world evidence on the integration of RCM into routine dermatologic practice and support its role as a second‐level imaging technique for the management of selected cutaneous lesions in specialized clinical settings.

## Graphical abstract

Integrated dermoscopy and reflectance confocal microscopy (RCM) enabled real‐world lesion stratification, demonstrated significant diagnostic concordance, and supported melanoma detection and management in a tertiary referral cohort.

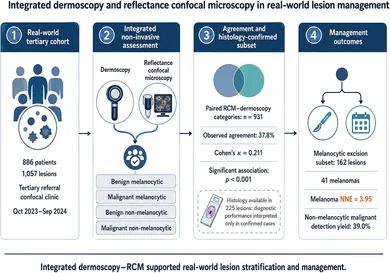

## 1 Introduction

Skin cancer represents a major and growing public health burden worldwide. Cutaneous malignancies are broadly classified into non‐melanoma skin cancers (NMSC), mainly basal cell carcinoma (BCC) and squamous cell carcinoma (SCC), and melanoma. NMSCs are the most frequently diagnosed malignancies globally, with a steadily increasing incidence driven by population aging, cumulative ultraviolet exposure, and improved detection [1, 2, 3, 4, 5]. Melanoma is less common but remains the leading cause of skin cancer‐related mortality; therefore, early diagnosis remains essential. In routine practice, clinicians must balance prompt identification of malignancy with avoidance of unnecessary excision of benign lesions. Dermoscopy is the cornerstone of non‐invasive skin lesion assessment and improves diagnostic sensitivity compared with naked‐eye examination [6, 7]. Standardized terminology and validated algorithms have strengthened the evaluation of melanoma and other cutaneous tumors [8, 9, 10, 11, 12, 13, 14, 15]. Nevertheless, dermoscopy may show reduced specificity in equivocal or feature‐poor lesions, particularly among melanocytic proliferations lacking classical diagnostic criteria, potentially leading to avoidable surgical excisions [16, 17]. This trade‐off is especially relevant in referral populations enriched for difficult lesions, where the clinical value of second‐level imaging depends not only on diagnostic performance but also on its impact on management.

Reflectance confocal microscopy (RCM) enables real‐time, in vivo visualization of the epidermis and superficial dermis at near‐histological resolution [18, 19, 20, 21]. By providing architectural and cytomorphological information, RCM complements dermoscopy and has shown a correlation with dermoscopic and histopathological features [19, 22, 23, 24, 25, 26, 27, 28, 29]. The combined use of dermoscopy and RCM has been associated with improved diagnostic refinement, fewer unnecessary excisions, and lower numbers needed to excise (NNE) values in specialized settings [29, 30, 31, 32, 33, 34]. Consequently, RCM has been incorporated into recommendations and consensus documents as an adjunctive diagnostic technique when available [35, 36, 37, 38].

However, several aspects of RCM use in routine practice remain insufficiently characterized. Most available studies focus on diagnostic accuracy in selected settings, whereas real‐world evidence linking RCM and dermoscopy to histological verification, management decisions, anatomical site, device utilization, and non‐melanocytic lesions is more limited [39, 40]. Beyond diagnostic categorization, these dimensions determine whether RCM meaningfully changes clinical pathways, supports conservative monitoring when appropriate, and remains reproducible outside highly selected research cohorts. This issue is clinically relevant because RCM is used not only to assess equivocal melanocytic lesions but also to support management of keratinocyte carcinomas, margin‐sensitive tumors, and lesions located in anatomically complex sites. This study aimed to evaluate the combined use of dermoscopy and confocal microscopy in a tertiary referral setting. Specifically, we investigated RCM–dermoscopy association and agreement across four diagnostic categories—benign melanocytic, malignant melanocytic, benign non‐melanocytic, and malignant non‐melanocytic—quantified management outcomes including melanoma NNE and malignant non‐melanocytic detection yield, and explored the influence of anatomical site, confocal device utilization, and temporal management patterns.

## 2 Materials and Methods

### 2.1 Study Design and Population

This retrospective observational study was conducted at the specialized confocal microscopy outpatient clinic of the Istituto Dermopatico dell'Immacolata (IDI), Rome, Italy. **C**onsecutive patients undergoing reflectance confocal microscopy (RCM) between October 2023 and September 2024 were considered eligible. A total of 886 patients, comprising 1057 cutaneous lesions, were evaluated using combined dermoscopy and RCM in routine clinical practice. Lesions were included in each analysis according to the availability of the required clinical, imaging, management, or histological variables. Lesions managed surgically underwent histopathological examination. Conservatively managed lesions were followed clinically, dermoscopically, and/or by RCM, in accordance with routine practice. Because histology was not systematically available for all lesions, analyses performed in the overall cohort were interpreted as agreement, association, diagnostic‐distribution, or management analyses, not as whole‐cohort diagnostic accuracy analyses. Diagnostic performance estimates were restricted to the histologically confirmed subset. Anatomical sites were grouped as trunk, upper limbs, lower limbs, head and neck, and genital region.

### 2.2 Diagnostic Procedures and Clinical Management

RCM imaging was performed using the VivaScope 1500 and VivaScope 3000 devices (MAVIG GmbH, Munich, Germany), according to the manufacturer's recommendations and standardized acquisition protocols routinely used at the study center.

RCM assessments were performed by dermatologists experienced in confocal microscopy and recorded using four diagnostic categories: benign melanocytic, malignant melanocytic, benign non‐melanocytic, and malignant non‐melanocytic. Benign melanocytic lesions were interpreted as benign melanocytic proliferations, whereas malignant melanocytic lesions were considered suspicious for melanoma. Benign non‐melanocytic lesions lacked features suspicious for malignancy, whereas malignant non‐melanocytic lesions were considered suspicious for NMSC or other malignant non‐melanocytic tumors. When adjudication was required, discordance was defined as disagreement in assignment to one of these four categories and was resolved by consensus among experienced readers. The retrospective database recorded final consensus categories rather than individual reader‐level assessments; therefore, formal interobserver agreement among RCM readers could not be calculated. Dermoscopy images were acquired under routine clinical conditions using standardized dermoscopic devices. Dermoscopic classification used the same four‐category framework and was compared with the RCM classification. Individual reader‐level dermoscopic assessments were not available in the retrospective database; therefore, formal per‐reader dermoscopic agreement could not be assessed. Clinical management reflected real‐world decision‐making and was not guided by a prespecified algorithm. Decisions integrated clinical examination, dermoscopy, RCM findings, anatomical site, patient history, feasibility of follow‐up, and physician judgment. Management strategies were categorized as surgical excision, RCM follow‐up, dermoscopic follow‐up, or clinical monitoring without advanced imaging. Histopathological diagnosis, when available, was classified using the same four‐category framework. Lesions without histological confirmation were not considered to have definitive ground truth.

### 2.3 Statistical Analysis and Ethics

Categorical variables were summarized as counts and percentages. The association between RCM and dermoscopic diagnostic distributions was evaluated using contingency tables and chi‐square tests; this association was not interpreted as agreement. Agreement was quantified using observed agreement and unweighted Cohen's kappa. Discordant diagnostic pairs were summarized to describe the direction and distribution of disagreement across the four categories. Associations between anatomical site, management strategy, RCM diagnostic category, and device utilization were assessed using chi‐square tests when the assumptions were met. Cramér's V was reported as an effect‐size measure where appropriate. Because genital lesions represented a small subgroup with potentially different management patterns, sensitivity analyses were performed after excluding genital lesions. The melanoma NNE was calculated in the dedicated melanocytic excision subset as the number of surgically excised melanocytic lesions divided by the number of histologically confirmed melanomas. Because NNE is a melanoma‐focused metric, malignant non‐melanocytic detection yield was calculated separately as the proportion of histologically confirmed excised non‐melanocytic lesions diagnosed as malignant non‐melanocytic tumors. In the histologically confirmed subset, confusion matrices compared RCM and dermoscopy with histopathology, and diagnostic performance metrics were interpreted in light of verification bias. Temporal trends in management were explored using monthly management proportions and regression‐based analyses; these were considered descriptive and not intended to infer causality or changes in diagnostic expertise. A two‐sided *p* value < 0.05 was considered statistically significant. Analyses were performed using IBM SPSS Statistics version 28.0. The study used fully anonymized retrospective data collected as part of standard care and was conducted in accordance with the Declaration of Helsinki. Under applicable Italian regulations for anonymized retrospective observational studies, formal ethics committee approval and additional written informed consent were not required.

## 3 Results

### 3.1 Cohort Characteristics and Histopathological Availability

The study included 886 patients and 1057 lesions evaluated in a real‐world tertiary referral setting. The cohort comprised 314 males (35.4%) and 572 females (64.6%), with a mean age of 49.9 years (range, 0–94). Paired RCM and dermoscopic diagnostic categories were available for 931 lesions (88.1%), which constituted the cohort for agreement analysis. Histopathological confirmation was available for 225 lesions (21.3%), whereas 832 lesions (78.7%) were managed without histology according to routine clinical practice. Among lesions with codable anatomical site (*n* = 1031), the most frequent locations were the trunk (421/1031, 40.8%) and head and neck region (375/1031, 36.4%), followed by lower limbs (152/1031, 14.7%), upper limbs (72/1031, 7.0%), and genital region (11/1031, 1.1%). Among lesions with available management data (*n* = 1002), management consisted of RCM follow‐up in 419 cases (41.8%), surgical excision in 331 (33.0%), clinical monitoring without advanced imaging in 196 (19.6%), and dermoscopic follow‐up in 56 (5.6%). Among lesions with available RCM category (*n* = 1020), 412 were benign melanocytic (40.4%), 230 malignant melanocytic (22.5%), 301 benign non‐melanocytic (29.5%), and 77 malignant non‐melanocytic (7.5%). Among lesions with available dermoscopic category (*n* = 942), 16 were benign melanocytic (1.7%), 640 malignant melanocytic (67.9%), 94 benign non‐melanocytic (10.0%), and 192 malignant non‐melanocytic (20.4%). Histology availability by RCM category was 12/412 (2.9%), 150/230 (65.2%), 20/301 (6.6%), and 43/77 (55.8%), respectively. Histology was available for 224/331 surgically excised lesions (67.7%) and for only 1 non‐excised lesion, indicating substantial verification bias and precluding estimates of whole‐cohort diagnostic accuracy (Table 1).

**TABLE 1:** Cohort characteristics, diagnostic distributions, management categories, and histological availability.

Variable	Category	Value
Cohort characteristics	Patients	886
	Lesions	1057
	Male sex	314/886 (35.4%)
	Female sex	572/886 (64.6%)
	Mean age	49.9 years
	Age range	0–94 years
Analysis availability	Paired RCM/dermoscopy diagnostic categories	931/1057 (88.1%)
	Histologically confirmed lesions	225/1057 (21.3%)
Anatomical site, *n*/*N* (%) among codable lesions	Trunk	421/1031 (40.8%)
	Upper limbs	72/1031 (7.0%)
	Lower limbs	152/1031 (14.7%)
	Head and neck	375/1031 (36.4%)
	Genital region	11/1031 (1.1%)
Clinical management, *n*/*N* (%) among lesions with available management data	Surgical excision	331/1002 (33.0%)
	RCM follow‐up	419/1002 (41.8%)
	Dermoscopic follow‐up	56/1002 (5.6%)
	Clinical monitoring without advanced imaging	196/1002 (19.6%)
RCM diagnostic categories, *n*/*N* (%)	Benign melanocytic	412/1020 (40.4%)
	Malignant melanocytic	230/1020 (22.5%)
	Benign non‐melanocytic	301/1020 (29.5%)
	Malignant non‐melanocytic	77/1020 (7.5%)
Dermoscopic diagnostic categories, *n*/*N* (%)	Benign melanocytic	16/942 (1.7%)
	Malignant melanocytic	640/942 (67.9%)
	Benign non‐melanocytic	94/942 (10.0%)
	Malignant non‐melanocytic	192/942 (20.4%)
Histology available by RCM category, *n*/*N* (%)	Benign melanocytic	12/412 (2.9%)
	Malignant melanocytic	150/230 (65.2%)
	Benign non‐melanocytic	20/301 (6.6%)
	Malignant non‐melanocytic	43/77 (55.8%)

*Note:* Percentages were calculated using the denominator available for each variable. Histology was not systematically available for all lesions and was obtained mainly from surgically managed lesions.

Abbreviation: RCM, reflectance confocal microscopy.

### 3.2 RCM–Dermoscopy Agreement and Histology‐Confirmed Analyses

Among 931 lesions with paired categories, RCM and dermoscopy were concordant in 352 cases (37.8%) and discordant in 579 (62.2%). Unweighted Cohen's kappa was 0.211 (95% CI, 0.172–0.251), indicating fair agreement. Diagnostic distributions were significantly associated (χ^2^ = 571.3, df = 9, *p* < 0.001; Cramér's V = 0.452), indicating a non‐random relationship between modalities but not high agreement. The dominant discordant pattern involved lesions classified as benign melanocytic by RCM and malignant melanocytic by dermoscopy. This subgroup included 354 lesions, corresponding to 38.0% of all paired cases and 61.1% of discordant cases. The reciprocal discordance, malignant melanocytic by RCM and benign melanocytic by dermoscopy, occurred in only three lesions, confirming a directional discrepancy rather than a row/column labelling error. Because histology was available for only a minority of lesions in the largest discordant subgroup, relative accuracy or specificity cannot be inferred for these cases. In the histologically confirmed subset, RCM could be compared with histopathology in 225 lesions. Four‐category observed agreement between RCM and histology was 37.8%, with Cohen's kappa of 0.219 (95% CI, 0.139–0.298). Dermoscopy could be compared with histopathology in 210 lesions, showing four‐category observed agreement of 35.7% and Cohen's kappa of 0.205 (95% CI, 0.125–0.285). Within the same selected subset, RCM–dermoscopy agreement was higher than in the overall paired cohort (79.5%; *κ* = 0.589; 95% CI, 0.480–0.699), reflecting enrichment for surgically managed suspicious lesions. For malignant non‐melanocytic lesions considered separately, RCM showed sensitivity of 84.4%, specificity of 91.7%, and accuracy of 90.7%, whereas dermoscopy showed sensitivity of 90.3%, specificity of 81.0%, and accuracy of 82.4%. These estimates were restricted to histologically confirmed lesions (Figure 1).

**FIGURE 1:**
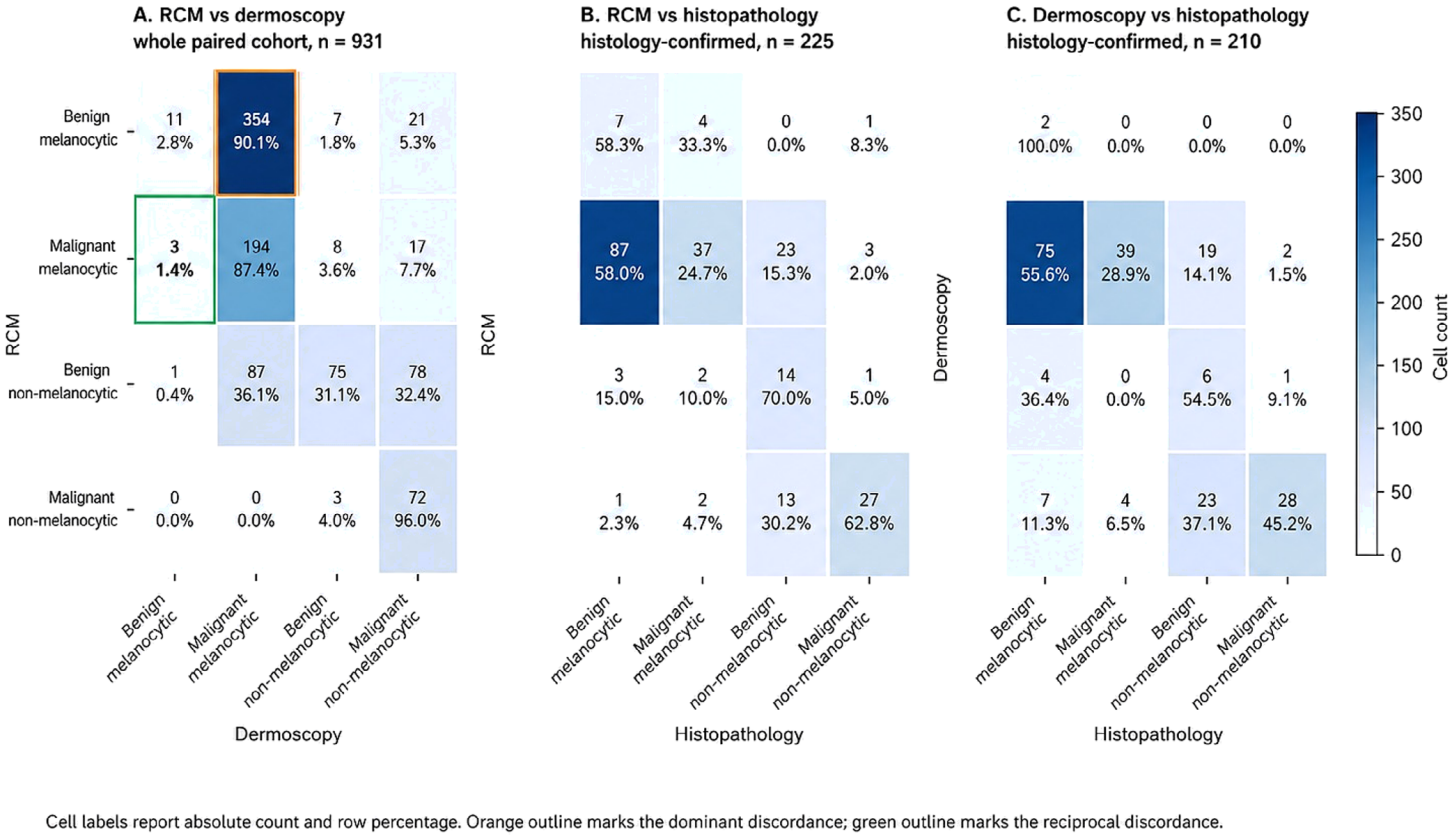
Diagnostic agreement and histology‐confirmed comparison. Multi‐panel heatmaps showing diagnostic category agreement across modalities. (A) RCM versus dermoscopy in the whole paired cohort with available paired diagnostic categories (*n* = 931). (B) RCM versus histopathology in histologically confirmed lesions (*n* = 225). (C) dermoscopy versus histopathology in histologically confirmed lesions (*n* = 210). Rows indicate the index modality and columns indicate the comparator modality. Each cell reports the absolute count and row percentage. The orange outline in panel A marks the dominant discordance, corresponding to lesions classified as benign melanocytic by RCM and malignant melanocytic by dermoscopy. The green outline marks the reciprocal discordance. RCM, reflectance confocal microscopy.

### 3.3 Management Outcomes, Anatomical Site, Device Use, and Temporal Analyses

Clinical management differed across RCM diagnostic categories. Lesions classified as malignant melanocytic were excised in 213/230 cases (92.6%), and malignant non‐melanocytic lesions in 68/77 (88.3%). Conversely, benign melanocytic lesions were predominantly managed with RCM follow‐up (283/412, 68.7%), while benign non‐melanocytic lesions were managed mainly with RCM follow‐up (119/301, 39.5%) or clinical monitoring without advanced imaging (123/301, 40.9%). Among the 77 RCM malignant non‐melanocytic lesions, seven were managed conservatively, including six with RCM follow‐up and one with clinical monitoring. Among 43 excised RCM malignant non‐melanocytic lesions with available histology, 27 were confirmed as malignant non‐melanocytic, corresponding to a category‐specific detection yield of 62.8% (Figure 2).

**FIGURE 2:**
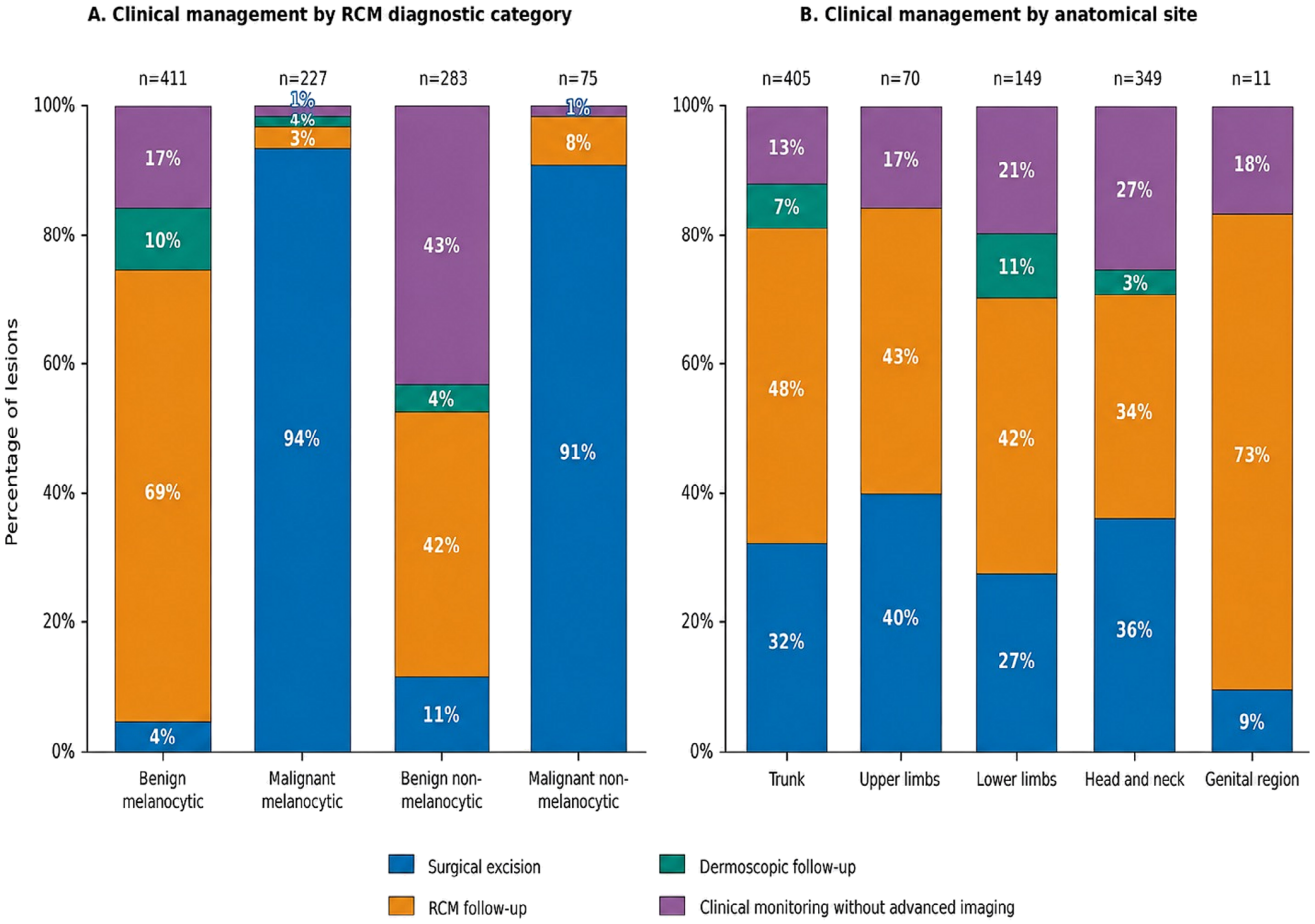
Clinical management according to the RCM diagnostic category and anatomical site. Stacked percentage bar charts showing management distribution according to (A) RCM diagnostic category, and (B) anatomical site. Management categories were not collapsed and include surgical excision, RCM follow‐up, dermoscopic follow‐up, and clinical monitoring without advanced imaging. Percentages are calculated within each RCM diagnostic category or anatomical site, respectively. Numbers above bars indicate the denominator for each category. RCM, reflectance confocal microscopy.

Among 162 melanocytic lesions considered suspicious after combined dermoscopic and confocal assessment and subsequently excised, histopathology confirmed 41 melanomas. The resulting melanoma NNE was 3.95. Because NNE is melanoma‐specific, non‐melanocytic malignancies were analyzed separately. Among histologically confirmed excised non‐melanocytic lesions, 32 were malignant non‐melanocytic tumors, corresponding to a detection yield of 39.0% and 2.6 non‐melanocytic excisions per malignant non‐melanocytic lesion detected. Agreement statistics, histology‐confirmed diagnostic performance, and management‐yield metrics are summarized in Table 2.

**TABLE 2:** Agreement statistics, histology‐confirmed diagnostic performance, and management‐yield metrics.

Section	Measure	Value
A. Agreement statistics	RCM vs. dermoscopy, whole paired cohort	*n* = 931
	Observed agreement	37.8%
	Cohen's *κ*	0.211
	95% CI	0.172–0.251
	Association test	*χ* ^2^ = 571.3, df = 9, *p* < 0.001
	Cramér's V	0.452
	RCM vs. histopathology	*n* = 225
	Observed agreement	37.8%
	Cohen's κ	0.219
	95% CI	0.139–0.298
	Dermoscopy vs. histopathology	*n* = 210
	Observed agreement	35.7%
	Cohen's κ	0.205
	95% CI	0.125–0.285
	RCM vs. dermoscopy within histologically confirmed subset	Observed agreement 79.5%
	Cohen's κ	0.589
	95% CI	0.480–0.699
B. Malignant non‐melanocytic endpoint, histologically confirmed subset	RCM	Sensitivity 84.4%; specificity 91.7%; accuracy 90.7%
	Dermoscopy	Sensitivity 90.3%; specificity 81.0%; accuracy 82.4%
C. Management‐yield metrics	Melanocytic excision subset	162 excised lesions
	Histologically confirmed melanomas	41
	Melanoma NNE	3.95
	Malignant non‐melanocytic detection yield among histologically confirmed excised non‐melanocytic lesions	39.0%
	Non‐melanocytic excisions per malignant non‐melanocytic lesion detected	2.6
	RCM malignant non‐melanocytic subgroup	27/43 confirmed; detection yield 62.8%

*Note:* NNE was calculated only in the melanocytic excision subset. Diagnostic performance metrics were restricted to histologically confirmed lesions. Detection yield for malignant non‐melanocytic lesions was reported separately and should not be interpreted as melanoma NNE. Agreement and diagnostic performance estimates should be interpreted in light of verification bias.

Abbreviations: CI, confidence interval; NNE, number needed to excise; RCM, reflectance confocal microscopy.

Clinical management distribution differed across anatomical sites (*χ* ^2^ = 52.9, df = 12, *p* < 0.001; Cramér's V = 0.134), although the association was weak. Surgical excision accounted for 31.1% of trunk lesions, 38.9% of upper limb lesions, 26.3% of lower limb lesions, 33.6% of head and neck lesions, and 9.1% of genital lesions. Exclusion of genital lesions did not materially change the overall excision rate (33.0% vs. 33.3%) or the associations between site and management (Cramér's V 0.134 vs. 0.128) or site and RCM category (0.276 vs. 0.275) (Figure 2).

RCM diagnostic distribution also differed by site (*χ* ^2^ = 228.5, df = 12, *p* < 0.001; Cramér's V = 0.276), with benign melanocytic lesions predominating on trunk and limbs and non‐melanocytic lesions more frequent on the head and neck. Device selection was strongly site‐driven: VivaScope 1500 predominated on trunk, upper limbs, and lower limbs, whereas VivaScope 3000 predominated on head and neck and genital sites (Cramér's V = 0.826) (Figure 3). Numerically higher RCM–histology concordance for VivaScope 3000 than VivaScope 1500 (46.0% vs. 33.3%) was considered exploratory because device allocation was confounded by site and diagnostic mix. Temporal analyses in 904 lesions with valid examination dates showed no significant trend in monthly surgical excision proportion (slope −1.5% per month, R^2^ = 0.19, *p* = 0.163).

**FIGURE 3:**
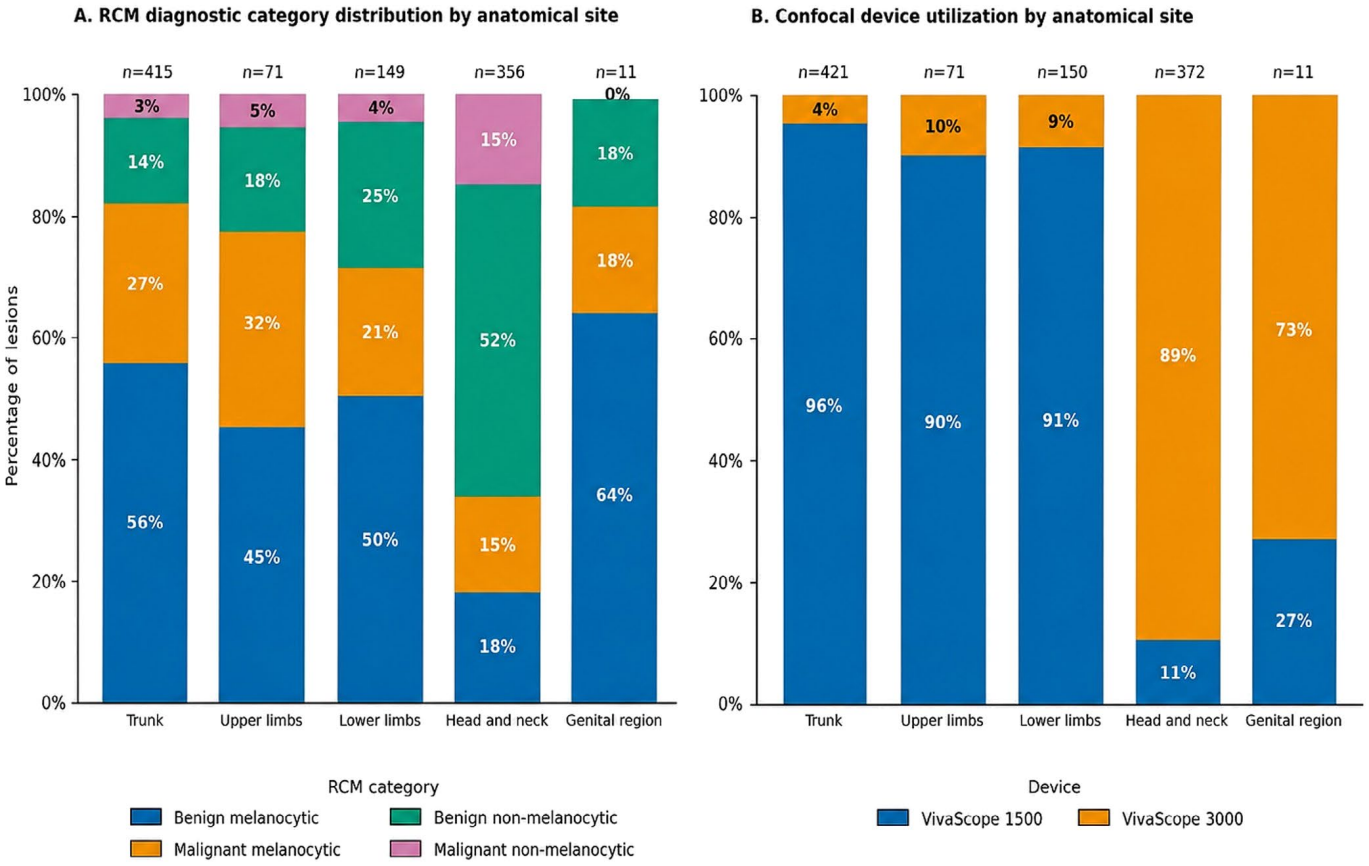
Anatomical site‐related diagnostic distribution and confocal device utilization. Stacked percentage bar charts showing (A) RCM diagnostic category distribution by anatomical site, and (B) confocal device utilization by anatomical site. Diagnostic categories include benign melanocytic, malignant melanocytic, benign non‐melanocytic, and malignant non‐melanocytic lesions. Device categories include VivaScope 1500 and VivaScope 3000. Percentages are calculated within each anatomical site. Numbers above bars indicate the denominator for each site‐specific analysis. RCM, reflectance confocal microscopy.

## 4 Discussion

This real‐world tertiary referral study shows that RCM integrated with dermoscopy was associated with clinically relevant lesion stratification and management, but it should not be interpreted as a whole‐cohort diagnostic accuracy study. RCM and dermoscopy showed significantly associated diagnostic distributions but only fair agreement, confirming that chi‐square association and kappa‐based agreement measure different constructs. The largest discordance involved lesions categorized as malignant melanocytic by dermoscopy and benign melanocytic by RCM. This finding should not be read as evidence of dermoscopic overcalling or RCM superiority, because systematic histological ground truth was unavailable for most discordant lesions. Instead, it reflects the case mix of a referral confocal clinic, where dermoscopy often identifies equivocal lesions requiring second‐level assessment, and RCM provides additional morphological information to support either excision or follow‐up. This is consistent with the role of RCM as an adjunct, not a replacement, for dermoscopy in equivocal lesions [29, 30, 31, 32, 33, 41, 42, 43]. Verification bias is the main interpretive constraint. Histology was obtained mainly for excised lesions and was substantially more frequent in malignant RCM categories than in benign categories. Therefore, sensitivity, specificity, predictive values, and accuracy cannot be estimated for the whole cohort. The histologically confirmed subset was enriched for suspicious lesions, explaining the higher RCM–dermoscopy agreement within this subgroup and limiting interpretation of binary malignant‐versus‐benign performance. Within this selected subset, both modalities contributed to the assessment of malignant non‐melanocytic lesions, with RCM showing higher specificity and accuracy, and dermoscopy showing higher sensitivity. These findings are clinically relevant but remain exploratory, because anatomical site, lesion type, clinical suspicion, and device selection were interrelated. The four‐category framework is important because malignant non‐melanocytic lesions were not marginal in clinical decision‐making. Lesions classified by RCM as malignant non‐melanocytic had a high excision rate and a 62.8% category‐specific histological detection yield among excised lesions with available histology. This broadens the study's message beyond melanoma and aligns with the recognized use of RCM for keratinocyte carcinomas and other non‐melanocytic tumors [44, 45]. The melanoma NNE of 3.95 suggests efficient melanoma detection among excised melanocytic lesions and is consistent with evidence that RCM can reduce unnecessary excisions in expert settings [29, 31, 32, 46, 47, 48].

However, this NNE derives from a high‐volume tertiary center with experienced readers and a surgically selected subset, not from an unselected screening population. It should therefore not be generalized to community practice or centers with different expertise. The separate malignant non‐melanocytic detection yield complements melanoma NNE but should not be conflated with it. Anatomical site influenced the distribution of diagnostics, management, and device use. Benign melanocytic lesions predominated on trunk and limbs, whereas non‐melanocytic lesions were more frequent on the head and neck, consistent with actinic damage and keratinocyte carcinoma burden in chronically sun‐exposed areas. Management associations were statistically significant but weak, and sensitivity analysis excluding genital lesions supported robustness despite the small genital subgroup. VivaScope 3000 was preferentially used at anatomically complex sites, reflecting practical workflow rather than intrinsic diagnostic superiority [39, 40]. Although RCM may also assist with preoperative assessment and margin mapping in selected lesions, including lentigo maligna and lentigo maligna melanoma [36, 39, 49], this was not specifically evaluated here. Temporal analyses were descriptive and did not support a significant reduction in excision rate during the study period. The study's strengths include its large real‐world cohort, a consecutive tertiary referral setting, a four‐category diagnostic framework, integration of management outcomes, histology‐confirmed subset analyses, melanoma NNE, malignant non‐melanocytic detection yield, and a sensitivity analysis excluding genital lesions. These elements directly address several limitations of studies focused only on melanoma accuracy endpoints. Limitations include the retrospective single‐center design, tertiary referral selection, lack of systematic histology, absence of individual reader‐level data, non‐algorithmic management, confounding by site, device, and lesion type, small genital subgroup, and exploratory temporal analyses. Overall, this study describes how combined dermoscopy and RCM function within expert clinical workflows rather than establishing population‐level diagnostic accuracy. Its value lies in linking diagnostic classification to actual management, while explicitly separating verified diagnostic performance from unverified real‐world follow‐up pathways. This distinction is essential when interpreting studies in which histology is appropriately reserved for lesions selected for excision in daily practice.

## 5 Conclusions

In this real‐world tertiary referral cohort, RCM integrated with dermoscopy supported lesion stratification and management across melanocytic and non‐melanocytic categories. The significant association but fair agreement between modalities supports their complementary role in second‐level assessment. The melanoma NNE of 3.95 and malignant non‐melanocytic detection yield indicate efficient management within a high‐expertise setting, but selective histological verification and the tertiary context limit generalizability. Until such data are available, these findings should be interpreted as evidence of clinical utility within expert workflows rather than as population‐level diagnostic accuracy. Prospective multicenter studies with standardized algorithms, systematic outcome verification, and broader clinical settings are needed to define reproducibility.

## Funding

The authors have nothing to report.

## Ethics Statement

This study was conducted in accordance with the principles of the Declaration of Helsinki. The study consisted exclusively of a retrospective analysis of fully anonymized data routinely collected as part of standard clinical care, with no study‐specific procedures or interventions. According to applicable Italian regulations governing retrospective observational studies based on anonymized data, formal review and approval by an Institutional Review Board or Ethics Committee were not required.

## Consent

Informed consent for diagnostic procedures was obtained as part of routine clinical practice. For this retrospective analysis based on anonymized data, additional written informed consent was not required.

## Conflicts of Interest

The authors declare no conflicts of interest.

## Data Availability

The data that support the findings of this study are available from the corresponding author upon reasonable request.
